# Acceptance and Commitment Therapy for Health Behavior Change: A Contextually-Driven Approach

**DOI:** 10.3389/fpsyg.2017.02350

**Published:** 2018-01-11

**Authors:** Chun-Qing Zhang, Emily Leeming, Patrick Smith, Pak-Kwong Chung, Martin S. Hagger, Steven C. Hayes

**Affiliations:** ^1^Department of Physical Education, Hong Kong Baptist University, Kowloon, Hong Kong; ^2^Department of Psychology, University of Nevada, Reno, Reno, NV, United States; ^3^School of Psychology and Speech Pathology, Faculty of Health Sciences, Curtin University, Bentley, WA, Australia; ^4^Department of Sport Sciences, University of Jyväskylä, Jyväskylä, Finland

**Keywords:** ACT, behavior change, behavior maintenance, psychological flexibility, relational frame theory

## Abstract

Promoting health behavior change presents an important challenge to theory and research in the field of health psychology. In this paper, we introduce a context-driven approach, the Acceptance and Commitment Therapy (ACT) model which is built on Relational Frame Theory. The ACT-based intervention aims to promote individuals’ new health behavior patterns through the improvement of the key construct of psychological flexibility, which is defined as the ability to contact the present moment more fully with acceptance and mindfulness as a conscious human being. Building on the psychological flexibility model, implemented through the six core ACT processes, individuals improve maintenance of long term health behavior change through committed acts in service of chosen values while acknowledging and accepting the existence of contrary thoughts, rules, and emotions as part of themselves but not determinant of their behaviors. Taking advantage of this context-driven approach of health behavior change, we recommend researchers and practitioners to design their health behavior change intervention programs based on ACT.

## Introduction

The pragmatic utility of psychological interventions on health behavior changes are judged by their effectiveness in promoting sustained and desired behavior change over an extended period of time. For example, effective interventions are not only those that get people to increase their steps per day or to eat a healthy meal, but also are those that maintain such gains months or years beyond the initial intervention. Mainstream health behavior change approaches have focused primarily on the content of cognitive and emotional variables that are thought to support long-term behavior change. Traditionally, health behavior change interventions target the social cognitive and belief-based variables to increase individuals’ intention (i.e., a person’s motivation toward the target behavior in terms of direction and intensity) and self-efficacy (i.e., one’s confidence in being capable of performing a novel behavior) in the hopes of maintaining the health behavior change (Schwarzer, 2008). While these approaches can be helpful to a degree, the magnitude of health problems and the maintenance of health behavior change suggests that alternatives are needed (Kwasnicka et al., 2016). One important approach that gains more and more momentum is to apply the context-driven approach to understand and predict health behavior change (Hayes, 2004). In the current mini review, the authors aim to introduce the context-driven approach of Acceptance and Commitment Therapy (ACT; Hayes et al., 1999) along with the description of how and why Relational Frame Theory (RFT; Hayes et al., 2001) and psychological flexibility provide coherent theoretical foundations and validated measures for ACT-based health behavior change. This should not be viewed as a detailed guidance and/or a systematic and comprehensive review, but more of a brief explanation and introduction focused on illustrating the links between the basic principles of ACT and related health behavior change.

As compared to the content-driven approach, in which behavior change is based on thought content unique to each particular behavior (Ellis, 1962; Beck, 1976), a context-driven approach examines the social, psychological, and situational context that regulates the behavioral impact of thought and emotion (Hayes et al., 1999). For health behavior change, instead of trying to directly change difficult thoughts or feelings, acceptance and mindfulness-based skills can be cultivated to foster greater behavioral regulation. Perhaps the most studied set of contextual processes of this kind is psychological flexibility, which is “the ability to contact the present moment more fully as a conscious human being and to change, or persist in, behavior when doing so serves valued ends” (Biglan et al., 2008). Psychological flexibility is *contextual* in the sense that it refers to individuals changing their *relationships* with private events (i.e., thoughts, memories, feelings, and bodily sensations), not the events themselves. For example, psychological flexibility may focus on teaching a dieter to be mindful and observe an urge to eat a chocolate cake without necessarily attempting get rid of that urge. The content surrounding a temptation to eat something delicious may include such things as judgments about whether this urge is good or bad; psychological flexibility suggests that the focus should be on how the individual interacts with these thoughts, rather than their form or frequency. Accordingly, the ultimate goal for individuals, initiating and maintaining the health behavior change, is to make said change(s) consistent with their chosen values (e.g., having a healthy lifestyle) even in the face of difficult thoughts or emotions.

Health behavior change is a dynamic process. Psychological flexibility has much to offer in the context of health behavior change as a theoretical guide for cultivating long-term improvements in behavior. According to Kashdan and Rottenberg (2010), four aspects of psychological flexibility can be viewed as fundamental to health, including: (a) recognizing and adapting to various situational demands, (b) shifting mindsets or perspective when personal or social functioning are compromised, (c) balancing competing desires, needs, and life domains, and (d) being aware of, open, and committed to behaviors that are congruent with deeply held values. These four aspects of psychological flexibility are well positioned to explain successful behavioral maintenance toward healthy living in a real-life context (Kwasnicka et al., 2016).

In principle, psychological flexibility can facilitate lasting change in three ways: (a) by increasing commitment to, and improved maintenance of, value-driven behaviors, (b) by strengthening a willing, open, and accepting method of experiencing psychological events thus reducing psychological barriers to behavior change, and (c) by improved awareness of one’s internal and external environment through mindfulness processes that allow behavioral choices to be better fitted to the contextual situation (Butryn et al., 2011). Functionally speaking, these processes are assessed empirically, in part, by examining whether psychological flexibility serves as the change mechanism for maintenance of adaptive and healthy behaviors (Ciarrochi et al., 2010).

Acceptance and Commitment Therapy is a behavior change method based on RFT and is explicitly oriented toward the development of greater psychological flexibility (Hayes et al., 1999). Although much of the early ACT work targeted mental health, from the beginning there was also a focus on health behavior change (of the first three studies done on ACT in the 1980s one was on diet, and another on dealing with tolerance of physical pain) and that interest has only grown in recent years (e.g., Butryn et al., 2011; Manlick et al., 2013; Bricker et al., 2014, 2017; Moffitt and Mohr, 2015). ACT offers an alternative to traditional attempts to control unwanted psychological experiences. Rather than trying to control the content of thinking and emotions, ACT aims to help individuals change their relationship to these events (Hayes, 2004). In ACT, the goals of the health behavior change interventions are not explicit replacement of previous unhealthy psychological events with new and healthy events, but the concurrent cultivation of acceptance toward of the occurrence of unhealthy psychological events, defusion from strict adherence to those events (i.e., observe the events for what they are as just thoughts of our mind, rather than becoming entangled and fused with them), and the committed action of behaviors that support living in ways that serve predetermined healthy values. In this way, habits for the new healthy behaviors may be established with greater resiliency to psychological barriers.

Preliminary evidence of ACT on direct and initial behavior change as well as promotion of behavioral maintenance has been established. ACT has been investigated in several health related domains, with positive long-term results. For example, the effectiveness of a randomized brief physical-activity-focused ACT-based intervention produced significant increases in individuals’ levels of physical activity. The skills taught in this intervention were mindfulness, values clarification, and willingness to experience distress via face-to-face intervention (Butryn et al., 2011) or via DVD (Moffitt and Mohr, 2015). In smoking cessation treatment, the effectiveness of ACT as compared to other interventions (e.g., nicotine replacement treatment, functional analytic psychotherapy, and cognitive behavioral therapy) has been demonstrated in a series of RCTs (e.g., Gifford et al., 2004, 2011; Hernández-López et al., 2009; Bricker et al., 2014, 2017). ACT has been recommended as an acceptance-based self-regulation framework for weight management (Lillis and Kendra, 2014), with awareness of decision-making thoughts and commitment to chosen values viewed as two key components (Forman and Butryn, 2015). For example, a 1-day mindfulness and acceptance-based workshop targeting obesity-related stigma and psychological distress is effective on weight loss and weight-specific acceptance coping; the intervention effects on weight loss was also found mediated by psychological flexibility (Lillis et al., 2009).

## Relational Frame Theory and Health Behavior Change

As the underlying foundation of the ACT, RFT is a contemporary behavioral account of language and human cognition. RFT claims that language is not based on learned associations or direct contingencies of a typical variety but is rather based on learned relational responses – patterns of responding to one event in terms of another (Hayes et al., 2001). Taking in to the context of health behavior change, RFT provides advantages in the degree of precision made possible when analyzing verbal contributions to complex human behavior. When applied to the topic of health related behaviors, it provides a preliminary behavioral account for how specific verbal rules come to exert control over responding (Barnes and Keenan, 1993; Carpentier et al., 2002). For example, the sound of the word “cigarette” is placed in a “frame of coordination” or “sameness” with a thin cylinder of finely cut tobacco rolled in paper. From an RFT perspective, this relational response is not dependent on the sound of the name because it is under arbitrary contextual control, not the form of the related events or even direct contact with them (Hayes, 1994; Barnes-Holmes and Barnes-Holmes, 2000; Hayes et al., 2001). The word “is” in the sentence “this is a cigarette” regulates the relational response of sameness between the name and object. These mutual relations then combine into networks of relations and the effects of related events are transformed. If you were told that a particular brand of cigarettes is laced with poison, you might be afraid to touch a cigarette of that brand even though you had no direct experience of bad things happening. From an RFT point of view, health behavioral change maintenance depends on not only one triggering factor like the intention of doing the behaviors but strengthening or weakening existing relational responses and learning new relational responses in the context of the target behavior(s).

Relational Frame Theory also provides grounds for considering contextual forms of ACT-based interventions versus content-based forms. If cognitive relations are learned, it is not fully possible to remove the historically established psychological relations between environmental cues and past unhealthy behaviors because there is no psychological process called “unlearning” (Hayes, 2004). Furthermore, if relating can be based on arbitrary cues it is hard to imagine how to deliberately alter ones cognitive networks without unexpected effects. This makes lasting change difficult. For example, telling yourself not to think of eating unhealthy food (say “don’t think about potato chips”) may paradoxically increase the likelihood that you are thinking about unhealthy food, since the rule contains the very verbal stimuli (the words “potato chips”) that are related to the unhealthy food, and every time you check to see if you are following the rule, you are very close to violating it (Wegner, 1994). This paradox of emotional or cognitive control may help explain why attempting to control unhealthy behaviors may at times cause the inverse effect and why even individuals who choose to change their eating behavior, from an unhealthy to a healthy diet, may struggle to maintain this new lifestyle choice despite reporting high motivation.

To overcome the problem of this paradoxical effect, RFT provides a theoretical explanation for the importance of using ACT-based interventions to develop psychological flexibility toward the verbal/cognitive networks that establish relations among stimuli, rules, and behaviors. People may need to learn how to strengthen or weaken the behavioral impact of rules rather than attempt to relate their presence or absence to success or failure. This breath of application is one reason ACT has been used to promote individuals’ psychological flexibility across a wide domain of health-related behaviors (e.g., Lillis et al., 2009; Butryn et al., 2011). For example, a diabetic person who can strengthen a values-linked rule such as “if I carry too much excessive weight I may not see my children grow up” may successfully reduce excessive eating throughout the day. Conversely, learning to weaken the impact of shame-linked rules like “I am a fat failure” through mindfulness and defusion methods described below fosters that same behavioral end.

Very recently, implicit methods of cognitive assessment, derived from RFT, have been used to predict the motivational impact of verbal descriptions on the relation between athletic activity and its outcomes, altering exercise levels and persistence through motivational operations (Jackson et al., 2016). Accordingly, the effects of the sensory, or perceptual, consequences of eating can be altered based on how an individual *frames* those consequences verbally, and by how the person relates to their own verbal processes. A similar effect has been empirically shown in the physical activity of people who avoid pain (e.g., Vowles et al., 2007). Therefore, more research should be conducted in the fields of health, behavior, and clinical psychology to further clarify the processes underlying RFT principles, ACT processes, and health behavior change.

## Act for Health Behavior Change: The Psychological Flexibility Model

Psychological flexibility model is a behavior change model based on RFT and its applied extensions that is used for understanding how rule-following behavior can affect behavior (Hayes et al., 1999; Gross and Fox, 2009) based on how people interact with their own language processes (Bond et al., 2006). According to the psychological flexibility model, which underpins ACT, psychological flexibility consists of six primary components: defusion, acceptance, self as context, contact with the present moment, values, and committed action. Psychological inflexibility is the opposite: fusion, experiential avoidance, the conceptualized self, rigid attention to the past or future, unclear values, and inaction, impulsivity, or persistence avoidance.

Psychological flexibility promotes behavior that aligns with the individual’s values rather than allowing thoughts about events to dominate regardless of their usefulness. To date, ACT is the most researched intervention model targeting psychological flexibility. Psychological flexibility is described as the ability to contact the present moment more fully as a conscious human being, and to change or persist in behavior such that one continues to behave in a way that is consistent with their pre-established and identified values (Hayes et al., 1996). The following will briefly describe these six flexibility processes and relate them to health behavior.

### Defusion

A defining concept of psychological flexibility is defusion. In situations where fusion occurs, individuals respond to the content of their language as if those descriptions are literally occurring (Hayes et al., 1999). For example, an individual attempting to quit smoking may experiencing physiological distress and may engage in verbal behavior that describes the context as “this is too difficult, I can’t quit.” When this verbal behavior is seen as a literal description of ability, the individual may return to smoking as a function of the description of the event and not as function of what they are physically capable of achieving. Defusion is the use of function-altering cues and strategies to reduce the transformation of stimulus functions in such cases, thus changing the impact of verbal events on other behavioral processes. Said in a more commonsense way, defusion it looking at thoughts with an attitude of dispassionate curiosity. Defusion methods, such as repeating the name of a feared object until it loses meaning, may reduce the impact of such thoughts (Blackledge, 2015).

### Acceptance

Acceptance occurs when an individual willingly experiences automatic, and sometimes unwanted, emotions or sensations without attempting to control the form, frequency, or situational sensitivity of these experiences (Bond et al., 2006). The psychological flexibility model contends it is not the content of emotions that become problematic to a quality life but rather problems arise when individuals interact with these events in an avoidant way (Bond et al., 2006). An example of behavior that is not indicative of acceptance is when an obese individual, who experiences anxiety around exercise and the gym, avoids these situations where anxiety occurs. Conversely, an individual experiencing anxiety around exercise and engaging in acceptance responses would instead acknowledge the anxiety with a sense of dispassionate curiosity and allow themselves to experience these emotions and situations where they occur. Scores of studies have shown that such strategies increase task persistence (Levin et al., 2012).

### Flexible Attention to the Present Moment

Human beings are uniquely adept at problem solving and planning. While these behaviors are often beneficial, they can sometimes be maladaptive, especially when language patterns become fused with temporal and evaluative statements. Problem solving always requires examination of the past and future (e.g., “How did I get here?” “Where am I going?”), but can overwhelm flexible attention to the present environment, both external and internal. Working in coordination with acceptance and defusion, contact with the present moment helps individuals respond while in touch with current environmental demands rather than merely the “what if” contexts reflective of rumination over past experiences and anxious anticipation of future ones (Bond et al., 2006). When individuals attend flexibly, fluidly, and voluntarily to the immediately relevant internal and external environment, performance demands can be better adjusted to what is presently occurring.

### Self-as-Context

The concept of self-as-context refers to the perspective skills needed for an individual to report on their own behavior from a consistent perspective or point of view. RFT research shows three key deictic relational frames are involved: I/YOU, HERE/THERE, and NOW/THEN (Hayes et al., 2006). When “I/HERE/NOW” comes together, it yields a sense of self as an observer. An individual, who can see and recognize their own verbal descriptions of themselves as distinct from the “I/HERE/NOW” perspective, may feel less threatened by negative statements about the self. For example, someone engaging in addictive behavior stating “I’m a no good drunk” might more readily recognize that statement merely as a thought that does not summarize themselves as a totality. By changing the statement to “I am having the thought that I am a no good drunk” they may thus reorient toward better quality of life behaviors.

This first four flexibility processes provide a working definition of mindfulness in a psychological flexibility model. The processes of adopting chosen values and commitment to the action of health behaviors relates to both initiation and maintenance of health behavior change.

### Values

Values are defined as chosen immediate qualities of ongoing patterns of action that are verbally established as reinforcers (Hayes et al., 1999). For example, an individual may value “investing in family life.” Such a statement is differentiated from a goal in that a value is not a tangible outcome such as “Friday family movie night” but is instead a quality of action. Values are known to increase task persistence across multiple health related behavior (e.g., Chase et al., 2013; Jackson et al., 2016). An individual who values investing in family life will engage in multiple behaviors that contribute to that stated value, i.e., helping children with homework, attending soccer and dance lesions, date nights, open communication, dressing up for date nights, exercising to model and pattern healthy life behaviors and so on. Values based living then, is living in such a way that values provide direction like a compass.

### Committed Action

In clinical settings, committed action looks much like traditional behavioral approaches (Bond et al., 2006). As part of psychological flexibility, committed action takes the role of expanding an individual’s valued responses into larger and larger patterns of activity. Larger patterns can be built by obtainable intermediary goals that comport with pre-established values. For example, an individual who experiences exercise anxiety and has historically pulled out or stopped exercising, may now set the goal of walking for an hour, three times a week in alignment with their value of “living a healthy lifestyle through exercise.”

## Conclusion

Although ACT was originally developed in the field of clinical psychology (Hayes, 2004), it has shown promise in facilitating individuals’ health behavior change with greater efficacy and fulfillment in individuals’ real-life contexts. The authors call for the further application of ACT and its underlying psychological flexibility model into the promotion of health behavior change. In particular, the health behavior change of individuals with clinical conditions such as chronic pain in order to increase beneficial physical activity (e.g., VanBuskirk et al., 2014). Finally, we emphasize that empirical evidence, gathered through robust research designs, on the efficacy and resilience/durability of ACT interventions on health behavioral change is urgently needed (e.g., randomized controlled trials).

## Author Contributions

C-QZ, EL, PS, P-KC, MH, and SH contributed to the conception and writing of the content for the review.

## Conflict of Interest Statement

The authors declare that the research was conducted in the absence of any commercial or financial relationships that could be construed as a potential conflict of interest.
